# When people start getting real: The Group Living Skills Survey for extreme work environments

**DOI:** 10.3389/fpsyg.2024.1348119

**Published:** 2024-04-16

**Authors:** Lauren Blackwell Landon, Jennifer C. W. Miller, Suzanne T. Bell, Peter G. Roma

**Affiliations:** ^1^Behavioral Health and Performance Laboratory, Biomedical Research and Environmental Sciences Division, Human Health and Performance Directorate, KBR, at NASA Johnson Space Center, Houston, TX, United States; ^2^Behavioral Health and Performance Laboratory, Biomedical Research and Environmental Sciences Division, Human Health and Performance Directorate, JES Tech, at NASA Johnson Space Center, Houston, TX, United States; ^3^Behavioral Health and Performance Laboratory, Biomedical Research and Environmental Sciences Division, Human Health and Performance Directorate, NASA Johnson Space Center, Houston, TX, United States

**Keywords:** team, group living, scale development, extreme environment, spaceflight, measure

## Abstract

**Introduction:**

Group living skills (GLS), that is, being tidy and considerate of others, are an important skillset for teams who live and work together. However, this construct does not have a validated measure to enable an understanding of how group living skills influence team dynamics over time. We developed and validated a short measure of group living skills for teams living in extreme work environments.

**Methods:**

We collected data from 83 individuals in 24 teams living and working in space and spaceflight analog environments on missions of 45–240 days.

**Results:**

We provide evidence of reliability and validity for the GLS Survey over time and identify a two-factor structure. We also demonstrate its use as a measure of team-level dynamics and its utility as a sociometric measure to identify a person’s degree of group living skills.

**Discussion:**

We outline recommendations for using this new measure in future research and applied settings to understand this unique aspect of teams living and working together.

## Introduction

Group living skills (GLS) are a unique subset of workplace skills which apply to colleagues who live and work together. They are especially important for teams in isolated operational environments, such as spaceflight. Astronauts live and work together for many months. Similarly, other jobs require remote deployment with an intact crew, such as military operations, Antarctic research stations, and global ocean shipping. There are often physical, temporal, or psychological boundaries between work and personal life for each employee (Clark, 2000); however, in extreme situations, the line between work–life balance may blur substantially (Landon and Paoletti, 2021). For example, the crew in the small Orion space vehicle on the Artemis II mission will not have private crew quarters for sleeping or personal activities. The private crew quarters on the larger International Space Station (ISS)—viewed as a key psychological countermeasure by many astronauts—are only the size of a telephone booth. Even that small personal space is so highly valued that the NASA Astronaut Office requires private crew quarters for missions longer than 30 days (NASA, 2022; Dev et al., in press). The crew is always expected to be on-call for emergency response, and—obviously—they do not go home to family at night during a mission.

Living and working in extreme conditions can impact well-being, cohesion, and performance in a way that is not applicable to more typical workplaces. In the organizational behavior literature, the positive and reciprocal relationship between team cohesion and team performance is well established (Braun et al., 2020; Grossman et al., 2022). Work–life balance or work–non-work research often examines spillover effects from one area to the other. Conceivably, interpersonal interactions between isolated and confined crewmembers during non-work times may affect team cohesion and, ultimately, team and task performance and mission success. Isolation and confinement are also highly stressful (Schorn and Roma, 2021), potentially causing more negative moods and putting pressure on interpersonal relationships. Extended durations in such conditions also add a dose effect such that these deleterious effects increase over time. Astronaut’s journaling on the ISS, for example, report increased conflict over time and decreased group positivity, and journal entries describe conflict that is related to personal, not just work, items (Stuster, 2010, 2016). Mission simulation analog crews also engage in both social- and work-related conflicts (Bell et al., 2019; Marcinkowski et al., 2021) and have reported a decrease in positive affect and increased stress over time (Klein et al., 2023). Again, crews do not have the ability to take a break from the extreme environment, and living with messy, inconsiderate crewmembers adds to the stress. One ISS astronaut stated, “It is going to be a much, much, much different crew experience when you do not have a spaceship sufficiently large to ever get away from each other during the day” (Stuster, 2016).

Conversely, a crew with good group living skills may act as a support system for each other, buffering the negative effects of the stressful environment. These effects may be felt between individuals as dyads engage with each other in shared spaces and activities, or they may have influence at the team level as violating or following norms related to group living can affect the team climate and cohesion. Astronauts are trained on group living skills with the goal of enabling well-being and mission success (Landon et al., 2018; Landon and Paoletti, 2021; Sipes et al., 2021). It is important to measure and monitor all constructs of interest in the spaceflight environment to track skill development and deploy timely countermeasures. However, until recently, there was no direct measure of group living skills for an operational work environment. Researchers recommend disentangling teamwork from group living to distinguish unique elements for definition, measurement, and training (Landon and Paoletti, 2021). Our paper seeks to fill this measurement gap and enable the assessment and understanding of group living skills related to well-being, cohesion, and performance over time in extreme environments.

## The emergence of group living skills as a spaceflight competency

As the end of the 20th century neared, NASA renewed its emphasis on teamwork and group living for long-duration missions with diverse teams (Galarza and Holland, 1999). An exploratory study just prior to the 1990s Shuttle-*Mir* 4- to 6-month missions examined astronaut selection issues for long-duration missions with astronaut peer ratings (McFadden et al., 1994). Two clusters emerged as the researchers created performance measures: Group Living (i.e., teamwork, leadership, tolerance, difficulty, working on another 3-month mission with that person, and group living) and Job Competence (knowledge and job performance). The group living subfactor of the group living cluster included being a good listener, being considerate of others, being helpful, and tolerance toward others and their cultural differences. NASA completed seven missions to *Mir*, each with a single NASA astronaut living with two Russian cosmonauts. They realized there were many differences between long-duration *Mir* missions and the more typical two-week Space Shuttle missions of the previous two decades. Using the experiences of these *Mir* astronauts and findings from studies of analogous environments (e.g., Antarctic stations, submarines), NASA psychologists performed a job analysis for short- and long-duration mission profiles to prepare for the upcoming ISS missions (Galarza and Holland, 1999). The competency titled “Group Living Skills” was identified as the third most critical factor for long duration and the seventh most critical factor for short-duration missions. An updated job analysis in 2013 with ISS astronauts and psychological support personnel input distinguished group living skills as more important for long durations, placing it as the 13^th^ most important competency area for a 6-month ISS, sixth for a 12-month ISS, second for a 12-month communication-delayed mission with smaller vehicles, and third for a 36-month Mars-like mission (Barrett et al., 2015). In other words, group living skills become more salient in longer durations and increased isolation and confinement.

## Group Living Skills Survey development

### NASA team measures workshop and item selection

A group of spaceflight research and operations experts were invited to participate in a NASA workshop with the goal of creating a core set of team-oriented behavioral health and performance measures for operational environments (Roma et al., 2016). Identified measures included team cohesion, self-reported team performance, personality, and team process measures; these measures were also used in the data collections described below. Group living skills were identified as a unique construct or competency area without an adequate existing measure for the extreme operational environment of spaceflight. The distinct aspects of this competency area were reviewed and discussed by the experts at the measures workshop, referencing the work of previous astronaut job analyses that defined the group living skills competency area and provided some behavioral examples (McFadden et al., 1994 Galarza and Holland, 1999; Barrett et al., 2015). Consideration was also given to relevant research literature in psychology; studies from analogous populations such as deployed military; recent ISS mission crewmember journals, debriefs, and interviews; and NASA experts’ understanding of crew training and psychological conference information related to group living skills. There was an effort to trim away general teamwork aspects of group living that had historically been grouped together under the group living skills (GLS) label as they were viewed as a separate construct. Each GLS Survey item was selected to reflect unique aspects of a group living in an extreme environment. Finally, after follow-up discussions with NASA operational psychology experts for consensus on the preliminary set of items generated by the project team, a final set of six items was designated for inclusion in the GLS Survey (see Table 1). A free response item asks respondents to elaborate.

**Table 1:** Group living skills (GLS) Survey.

**Individual-level referent (or self-assessment) instructions**: Please rate your level of agreement with the following statements about [*selected teammate*] (if you are [*selected teammate*], please answer the questions about yourself)
**Clean and tidy with personal items**
**Clean and tidy with work items**
**Uses humor appropriately**
**Appreciates others’ knowledge, skills, and abilities**
**Considerate of others’ preferences**
If I were doing another mission, I would want [*selected teammate*] as a crewmate*
*Free response*: Please feel free to elaborate or provide details on any of the items above
**Crew-level referent instructions**: In your experience, please rate your level of agreement with the following statements for your entire crew:
**Clean and tidy with personal items**
**Clean and tidy with work items**
**Uses humor appropriately**
**Appreciates others’ knowledge, skills, and abilities**
**Considerate of others’ preferences**
If I were doing another mission, I would want this exact same crew*
*Free response*: Please feel free to elaborate or provide details on any of the items above

* indicates that the item was removed from the final five-item GLS Survey in later analyses and treated as a separate construct: team viability (scale anchors: 1 = Strongly Disagree, 4 = Neutral, 7 = Strongly Agree). Bold items were included in the final version of the GLS Survey.

### Item explanation

#### Clean and tidy with personal or work items

The first key aspect related to being a good “space roommate” is being tidy and picking up after oneself. There is substantial overlap in both mutual workspaces and mutual personal spaces as a crew lives and works in the same confined habitat. Simply keeping common areas clean and organized reduces stress in others as they have less clutter to sort through to find what they need and fewer distractions in their field of vision. Clutter can negatively impact well-being and productivity at home and work (Roster et al., 2016; Roster and Ferrari, 2020). Space habitats, especially space stations that have been occupied for multiple decades, such as the ISS, are cluttered. Stowage is an ongoing stressor, as numerous pieces of equipment are added to the station with each launch and upgrade, accumulating in translation paths and blocking areas intended for habitability and work (Gore et al., 2021). Clutter can be dangerous: hard-to-find equipment can delay addressing off-nominal events, or objects can float into a crewmember and cause injury (Greene et al., 2018). Tidiness meant to prepare a work area and maintain it for others is procedural, saving time onboard for mission objectives. Tidiness reduces spatial conflict of objects and reduces the interpersonal tensions that might arise from the owners of said objects. Stated one crewmember, “I pick up a little after them, but I am at the point now where I am leaving soon, and it is easier to let it go and let someone else worry about it. X leaves tools out and cables dangling in the middle of a module. Nothing big, but these little things are the difference between making work easy or hard” (Stuster, 2016, p. 64). Tidiness focused on personal items can ensure, for example, that odorous exercise clothes are tethered in agreed-upon areas so that smells are contained and expected. An individual may vary how they organize work versus personal items, but in a confined space, the lines between work and personal overlap. Importantly, decluttering and organizing have been identified as a skill, and training in such skills can have a lasting positive influence on managing clutter and behavioral health (Aso et al., 2017; Aso, 2018).

#### Considerate of others’ preferences

Consideration of others’ preferences stems from understanding that others’ backgrounds, cultures, personal needs, and situations are diverse. Having sensitivity to these issues requires empathy and perspective-taking cognitive abilities to identify and alter behaviors to accommodate other people, which can be a positive force in a cooperative team environment (Ku et al., 2015; Longmire and Harrison, 2018). Research indicates that dyads high on empathetic perspective-taking are more accurate in synchronizing actions, and empathetic perspective-taking enables anticipation of others’ behavior and improved coordination (Novembre et al., 2019). Key example issues to be aware of may include sensitivity to personal space and to typical routines or ways of working and engaging in personal activities. This item also captures an individual’s motivation to be open to different preferences and find out more about their crewmates. For multi-cultural crews, typical on ISS, cultural awareness and sensitivity are viewed by space psychology experts as particularly important to this aspect of group living. Past studies on Mir and the ISS have found evidence of national, occupational, and organizational cultural differences between American astronauts and Russian cosmonauts, which can complicate living and working together (Boyd et al., 2009). Multi-cultural crews add another layer of complexity to the complex system of spaceflight as crews navigate differences in hierarchies and power distance norms, collectivism versus individualism, gender norms, and how performance is assessed and rewarded in-mission (Bell et al., 2015; Käosaar et al., 2022). Others’ preferences referred to here can be broad as they encompass both work and non-work issues, which are reflected in the item phrasing.

#### Appreciates others’ knowledge, skills, and abilities

Cooperation and adaptability can also be supported by the appreciation of others’ knowledge, skills, and abilities for work and non-work actions. In a dangerous operational environment with a small crew, each individual is a critical part of supporting the crew’s health and safety and mission objectives. Teams are carefully composed to bring a unique and needed skillset to the mission. The team is also composed and trained so that they are able to deploy those skills efficiently and effectively together. Roles and responsibilities are carefully planned for mission tasks, but team members are also trained as backups or to provide proactive support when another crewmember acts as leader due to position or expertise (Landon et al., 2018). Pre-mission training as an intact team allows a transactive memory system to develop so everyone learns who knows what. A shared mental model develops for taskwork, teamwork, and the general norms of living and working together. Both forms of team cognition support task performance, affect within the team, and belief in team viability (Bachrach et al., 2019; Niler et al., 2021; DeChurch et al., 2023). Spaceflight is a high-consequence environment, so relying on others for safety is another way crews recognize the importance of complementary skillsets. Appreciation is ideally developed during pre-mission preparation periods, including training, and is reinforced as they accomplish tasks in flight and during team debrief opportunities. Interviews of NASA Shuttle astronauts indicated that skepticism related to a non-NASA career astronaut’s (i.e., payload specialist) predicted performance and integration into a crew was reduced when payload specialists were observed working hard, performing tasks successfully, and caring for other crewmembers (NASA, 2010). Appreciation can also stem from informal pre-mission interactions, highlighting the importance of intact and in-person (or virtual synchronous) events that allow for conversations and relationships to form between formal training time. Appreciation can be demonstrated formally and informally via specific recognition and symbols (e.g., awards) or by creating a culture where the team norms appreciative behavior and practices it consistently without prompting. The item is broadly written to recognize any type of appreciation for others’ knowledge, skills, and abilities that might be demonstrated by another crewmember.

#### Uses humor appropriately

Astronauts point to humor as an important way to form connections, alleviate stress and interpersonal tensions, keep connections strong, and maintain morale (Stuster, 2016 Lungeanu et al., 2023). The positive influence of humor has been documented in analogous contexts such as healthcare teams, firefighters, and the military (e.g., Mesmer-Magnus et al., 2012; Sliter et al., 2014; Vivona, 2014). The key to team-supportive humor is the appropriateness for that particular team and context, determined by the individuals giving and receiving jokes. It should be inclusive, rather than exclusive or meant to denigrate others in order for it to maintain team cohesion. A spaceflight analog study found that crewmembers with an affiliative style of humor used positive humor over time, while those with an aggressive style used more negative humor over time (Vazquez and Bell, 2023). Findings showed that the relationship between an affiliative humor style and use was stronger during unstructured, social activities (i.e., lunch) versus a structured, work-oriented decision-making task, suggesting humor is context-dependent. A past study on humor as a coping mechanism in spaceflight found that astronauts and cosmonauts used it more on long-duration flights, and cosmonauts used less affiliative humor than astronauts (Brcic et al., 2018). These findings suggest that cultural nuances and considerations of others are tied to the successful use of team-appropriate humor. The study also found that the use of self-deprecating humor decreased during the mission compared to pre- and post-flight, likely to maintain positivity and morale. Thus, humor is important for group living skills, but it must be deemed “appropriate” to be supportive of the team. The definition of “appropriate” is up to each crew member to decide which drives the phrasing of that survey item.

#### Doing another mission with same crewmate or crew

Long-term commitment to living with colleagues is salient in isolated, confined, long-duration work environments. The final item about going on another mission with a particular individual or crew captures aspects of long-term team viability, that is, “a team’s capacity for sustainability and growth for success in future performance episodes” (Bell and Marentette, 2011, p. 276). It is slightly different from the other five items on the survey because it is future-focused, while the other items consider behaviors a respondent observed being performed by another crewmember. This item is more speculative and asks respondents to imagine what living and working on another extreme mission might be like and—importantly—if they believe the other person or crewmember would contribute positively to the predicted scenario. This item targets the frame of mind in psychologically committing to each other and mission success on long-duration missions. Based on the sociometric approach used in Russian isolation studies (e.g., Vinokhodova et al., 2012), earlier versions of the item added duration details [e.g., 3-month space station tour (McFadden et al., 1994); 3-year mission (Lungeanu et al., 2023)], but duration on the GLS Survey is unnamed such that respondents can consider the unique context of their mission experience with that person or crew.

### Survey structure and instructions

A brief survey was created to enhance the feasibility and acceptability in an operational environment, which requires reducing the crew time burden for surveys as much as possible (Allen et al., 2022; Matthews et al., 2022). The survey can be framed with different referents as the target of the questions. For individual-level ratings, each respondent is instructed to answer the survey separately about each individual teammate (i.e., peer referent). The respondent must indicate the target before answering the items each time. For self-assessment, respondents also answer the survey about themselves (i.e., self-referent). For crew-level assessments, each respondent answers the survey about the entire crew considered together (i.e., crew referent).

## Methods

### Participants

Data were collected from 83 individuals in 24 teams living and working in space and spaceflight analog environments; sample demographics are summarized in Table 2. Data were collected in the ISS, the Human Exploration Research Analog (HERA), and the Scientific International Research In a Unique terrestrial Station (SIRIUS) spaceflight analog missions. Characteristics of these environments are summarized in Table 3.

**Table 2:** Sample demographics from astronauts and spaceflight analog astronaut-like crewmembers.

	*n*	No. of crews	Size of crew	Age Mean (SD)	Sex	Education (% advanced degrees)	Military Exp. *n*	ICE Exp. *n*
ISS	23	10	3–11	44.83 (7.10)	8F | 15 M	100%	–	–
HERA C4	16	4	4	40.12 (8.98)	6F | 10 M	75%	5	0
HERA C5	16	4	4	36.96 (5.68)	4F | 12 M	100%	6	2
HERA C6	16	4	4	37.47 (6.38)	8F | 8 M	100%	2	5
SIRIUS-19	6	1	6	34.34 (6.26)	3F | 3 M	100%	2	4
SIRIUS-21	6	1	6	33.63 (5.17)	3F | 3 M	100%	3	4
All	83	24	Analog = 4 Space = 7	39.42 (7.76)	32F | 51 M	95%	–	–

HERA, Human Exploration Research Analog; SIRIUS, Scientific International Research In a Unique Terrestrial Station; ISS, International Space Station; F, female; M, male; ICE Exp., experience in isolated, confined, extreme environments.

**Table 3:** Characteristics of study environments.

	Duration (days)	Size of crew	Approximate habitable volume	Private crew quarters	Multi-cultural crews	Comm. Delay (one-way)	Notable contextual factors
ISS	~190	3–11 (*M* = 7)	13,700 ft^3^ (four-bedroom house)	Yes	Always	Intermit-tent loss of signal	Actual spaceflight; occasional slam sleep shifts, 17 sunrises per day, crew rotation
HERA C4	45	4	5,200 ft^3^ (one-bedroom apt.)	Yes	Rarely	Up to 5 min	Asteroid rendezvous sim.; chronic sleep deprivation (5 h on weeknights)
HERA C5	45	4	4,700 ft^3^ with restricted airlock (one-bedroom apt.)	No	Rarely	Up to 5 min	Mars moon rendezvous sim.; permanently reduced privacy in HERA; 36-h sleep deprivation
HERA C6	45	4	4,700 ft^3^ with restricted airlock (one-bedroom apt.)	No	Rarely	Up to 5 min	Mars moon rendezvous sim.; increased autonomy; reduced privacy; 36-h sleep deprivation
SIRIUS-19	120	6	19,400 ft^3^	Yes	Always	Up to 5 min	One lunar landing sim; 36-h sleep deprivations
SIRIUS-21	240	5–6	19,400 ft^3^	Yes	Always	Up to 5 min	Three lunar landings sims.; 3, 36-h sleep deprivations; crewmember egress day 32
Common for all missions	Daily planning conferences with mission control; group meals; exercise; spaceflight-like food; operational tasks, EVAs (virtual or actual); named commander, crew roles; private medical, psychological, and family conferences; email access; recreational activities						

M, mean; Comm., communication; HERA, Human Exploration Research Analog; SIRIUS, Scientific International Research In a Unique Terrestrial Station; ISS, International Space Station; EVA, extravehicular activity; Sim., simulation.Habitable volume information from NASA Human Research Program’s Research and Operations Integration Element (personal communications, 2017–2023; Vessey et al., 2017; NASA, 2023a).

Measures of GLS, team performance, and team cohesion were collected as part of a larger behavioral health study protocol. Team cohesion was measured with six items (two for ISS), and team performance was measured with four items (one for ISS). Data collection frequency was dependent on the characteristics of the mission and environment (e.g., length, spaceflight vs. terrestrial analog). Given the length of the data collection period (2015–2023), there were also survey refinements (e.g., response format, addition of crew-level referent) made over time. Next, we describe the environments, protocols, and data harmonization process.

### ISS

#### Environment description

The ISS is in low Earth orbit, which carries with it the complexity of living and working in microgravity, a high-tempo workload, and a hostile environment with real danger. The typical six-month ISS crew of six astronauts experiences a crew rotation of three members approximately every 3 months (with occasional one- to two-week visitors), resupply and care packages, close coordination with experts in mission control (usually with real-time communication), readily accessible communication with family and friends on Earth, and meaningful work such as spacewalks or extravehicular activities (EVAs) and research tasks. Astronauts are rigorously selected to be team-oriented, resilient, adaptable, and self-sufficient (Landon et al., 2017; Beven et al., 2018; Schmidt and Spychalski, 2021). They are also selected for expertise such as advanced science/technology/engineering/mathematics (STEM) degrees, military and extreme environment experience, as well as passing a medical qualification and psychiatric screening. They undergo years of training in technical and team skills, including training and feedback in stress management, conflict management, teamwork, and group living skills (Landon et al., 2018; Sipes et al., 2021). An assigned crew will engage in an approximately 18-month pre-mission training program to prepare them for a specific mission together. While only some of that pre-mission training is with their intact crew, crewmembers often know each other for several years before the mission.

#### Study protocol

Astronauts on the ISS completed a digital version of GLS Survey approximately every 30 days as part of a spaceflight standard measures study (PI = G. Clement), only answering items with the crew-referent as the target. Measures of cohesion and performance (as well as other behavioral health and team measures) were completed at the same time. These are short forms of the measures used in the spaceflight analogs (i.e., HERA and SIRIUS) to reduce crew time burden (see Appendix A for items and Table 4 for psychometric properties).

**Table 4:** ICC(1) and ICC(2).

Measure	ICC(1)	ICC(2)	*N*
GLS all individuals (with self) and crew referents	0.25	0.96	1860
GLS peers	0.27	0.97	326
GLS crew referent	0.32	0.90	464
GLS roommate quality	0.55	0.99	326
Team cohesion	0.33	0.90	446
Team performance	0.11	0.69	448

For GLS, all individual, peers, and crew referents, the ICC(1) and ICC(2), were calculated from individual scores to examine the appropriateness of aggregating the scores to the team level and the reliability of the group mean. For Roommate Quality, ICC(1B) and ICC(2B) reflect the clustering of individual ratings on a particular target. N = the number of observations.

### HERA

#### Environment description

The HERA spaceflight simulation missions occur in a habitat at NASA Johnson Space Center in Houston, Texas (Vessey et al., 2017; Cromwell and Neigut, 2021). Each Campaign is a series of four 45-day missions with the same set of studies, tasks, and analog environment designs, with only a change of crew members between missions. Changes may occur between campaigns, such as studies added or subtracted, and the habitat, tasks, and stressor protocols may be altered. Thus, each HERA campaign is treated separately in the analyses. Crewmembers are selected to be astronaut-like (i.e., 30–55 years old, advanced STEM degrees, psychologically resilient, Class 3 flight physical, no disqualifying medical or psychiatric conditions), and crews are usually mixed gender (see Table 2). All campaigns aim to be spaceflight-like, with living and working conditions similar to the ISS (see Table 3). The crews undergo a two-week pre-mission training period on mission tasks and team skills. They coordinate with mission support personnel, but this is communication delayed up to 5 min one-way.

#### Study protocol

HERA participants completed the GLS Survey via tablet device every 10 days during the mission. Campaign 4 (C4) crewmembers did not complete the GLS Survey with the crew-level referent, but C5 and C6 crewmembers completed the next iteration of the GLS Survey with individual-level referents and the crew-level referents. All HERA crewmembers completed other behavioral health and team measures on approximately the same mission day (see Appendix A for items and Table 4 for psychometric properties). Participants completed the team cohesion and team performance measures daily in all campaigns and during all missions. Observations taken the same day as the GLS Survey were merged into separate datasets (e.g., GLS and team cohesion as one dataset, whereas GLS and team performance are another dataset) to retain the highest number of observations. The *n* for each dataset varies due to the removal of careless observations or missing at random datapoints. Team cohesion and team performance measurements taken on non-GLS Survey days were not included in this analysis.

### SIRIUS

#### Environment description

The Scientific International Research In a Unique terrestrial Station (SIRIUS) analog missions occur in the NEK chamber (*Nezemnyy Eksperimental’nyy Kompleks*) in Moscow, Russia (NASA, 2023b). Crewmembers are selected to be astronaut-like with similar medical and psychological guidelines to HERA, and the mission scenario is also spaceflight-like with a crew of six (see Tables 2, 3). The crews undergo a pre-mission training period of up to 8 weeks on mission tasks and team skills. The crews coordinate with mission support personnel similar to HERA, also with a communication delay of up to 5 min one-way. Some studies and tasks were altered, added, or removed between the two SIRIUS missions. Also, the durations of the two missions were significantly different (120 days, 240 days), creating more distinctness between them. For these reasons, SIRIUS-19 and SIRIUS-21 are considered separately in the analyses.

#### Study protocol

Due to the longer duration of the SIRIUS missions and concerns for crew time burden, the sampling frequency was changed to collect the GLS Survey every 20 days in-mission. All SIRIUS crewmembers completed the GLS Survey via tablet device with the individual-level referents and with the crew-level referent. They completed team cohesion and team performance measures and other behavioral health and team measures at approximately the same time (see Appendix A for items and Table 4 for psychometric properties). Participants completed the team cohesion and performance measures with an approximate sampling frequency every 3 days. Observations taken the same day as the GLS Survey were merged, and those close to the same day were utilized to fully represent each crew. The range of target administrations varied from measurements taken 4 days later; however, most administrations were within 2 days of the GLS Survey. As with HERA, each measure was merged into a separate combined dataset to address the removed careless observations and missing at random datapoints.

### Data harmonization and preliminary analyses

Data were collected across several years, with multiple survey refinements related to (1) the target about which the respondent was considering (i.e., individual-level referent when rating peers or self- vs. crew-level referent when rating the crew) and (2) the response scaling (i.e., bipolar 201-point visual scale vs. 7-point Likert scale). Initially, there were only individual-level referents. The crew-level referent was added when the survey was extended to ISS data collection because the astronauts do not traditionally evaluate the performance of other individual crewmembers and reduce crew time burden. Thus, HERA C4 had only individual-level referents, the ISS had only crew-level referents, and HERA C5 and C6 and SIRIUS-19 and -21 had both individual-level and crew-level referents. The individual HERA C4 individual-level assessments were aggregated with self-ratings for each team at each time point to create substitute crew-level scores. Analyses from HERA C5 and C6 compared these peer/self-aggregated crew-level scores with the scores directly assessing the group living with the crew-level referent. A paired *t*-test with a null hypothesis significance test (NHST) and an equivalence test via two one-sided tests (TOST) were performed with an alpha level of 0.05. These tested the null hypotheses that the true mean difference is equal to 0 (NHST) and the true mean difference is more extreme than −0.15 and 0.15 (TOST). The equivalence test was significant, *t*(55) = −2.94, *p* < 0.01 (mean difference = 0.01 90% C.I. [−0.075, 0.09]; Hedges’s g(rm) = 0.01 90% C.I. [−0.20, 0.23]). Thus, C4 substitute crew-level scores were used in further analyses alongside the directly assessed crew-referent scores in all other missions.

HERA C4 and C5, SIRIUS-19, and earlier ISS data employed a 201-point visual analog scale ranging from −100 to +100 while HERA C6, SIRIUS-21, and later ISS data used a 7-point Likert scale. Anchors at the lowest ends of the scale were “Strongly Disagree,” and anchors at the highest end of the scale were “Strongly Agree.” The Likert scale displayed each of the 7 points on the scale and prompted the respondent to select one. The VAS did not display any points on the scale between the lowest and highest ends and prompted the respondent to simply slide the response marker to any point on the line spanning the two ends of the scale. Data from the visual analog scale were binned to a 7-point scale, which did not meaningfully change the results, so the 7-point scale was used for all further analyses.

During the item generation process, the first five items were focused on group living skills, while the sixth GLS Survey item was a global estimate about wanting to do another mission with the same crewmate or crew. Subject matter experts (SMEs) involved in the process suggested that the sixth item would likely be treated separately from the group living behaviors; therefore, we first ran a few preliminary analyses to confirm that it should be treated separately. A hierarchical cluster analysis was used to assess the collinearity, redundancy, and clustering of the six items across all timepoints and research settings. Additionally, each research setting was evaluated separately. At all levels of analysis, item correlations did not exceed 0.75, suggesting multicollinearity was not present (Flora, 2018). Correlations also suggested no redundant items. Further redundancy analysis with an *R*^2^ cutoff of 0.80 demonstrated no item could be predicted from all other items. In all analyses, the “another mission” item was the least correlated and did not cluster consistently with the other five items. This confirmed the expert opinion from the survey developers and evidence from the literature that this item is capturing a distinct, future-oriented team viability construct, which is different from capturing lived experiences in the other five items of GLS Survey. Thus, we proceed to use the “another mission” item as a measure of team viability.

### Analytical strategy for demonstrating reliability and validity

We examined the reliability of the scores and the validity of the measure in two ways: (1) as a team-level measure of group living skills and (2) as a measure of the group living skills of a particular individual in a crew, which we refer to as Roommate Quality.

#### Team-level measure of group living skills

We examined the reliability and validity of a team-level measure of group living skills by (1a: All individuals with self) aggregating crew members’ ratings of all crewmembers including their self-rating to the team level (Chan, 1998), (1b: Peers) aggregating crew members ratings of all other crewmembers excluding their self-rating to the team level, and (1c: Crew Referent) examining the team mean of the responses to items with the group-level referent level.

To examine reliability at the item level, we calculated omegas as a measure of internal consistency (McNeish, 2018). To examine the appropriateness of aggregating the scores to the team level, we calculated the proportion of total variance attributable to team membership [i.e., intraclass correlation, ICC(1)]. We also calculated the reliability of the team mean ICC(2) (Chen et al., 2004; LeBreton and Senter, 2008). To examine the consistency of scores over time (i.e., reliability of change), we conducted variance decomposition analyses that included item, time (mission day), and individual, then again at the team level with item, time, and team/crew (Cranford et al., 2006).

We examined the construct validity and factor structure of the GLS Survey with a multi-level confirmatory factor analysis (CFA; Fabrigar et al., 1999; Mathieu and Taylor, 2006). We also tested for measurement invariance over time (Putnick and Bornstein, 2016; Winter and Depaoli, 2022). Finally, we examined the extent to which GLS scores were related to team cohesion, team viability, and team performance (Kozlowski, 2015; Tannenbaum and Mathieu, 2015; see Appendix A).

#### Roommate quality

We measured the reliability and validity as a “Roommate Quality” measure by aggregating the scores for each individual as rated by their other teammates to look at how each individual on a team was perceived by others. Our analyses followed a similar progression as the previous set of team-level analyses.

## Results

### Team-level measure of group living skills

#### Reliability analysis

For the GLS Survey, when all individuals (including self and crew) were included as referents, ICC(1) and ICC(2) were large [i.e., ICC(1) ≥ 0.25 (LeBreton and Senter, 2008)] and excellent [i.e., ICC(2) > 0.75 (Fleiss, 1986)], and the model had good internal consistency (total ω = 0.93; McNeish, 2018). For the GLS Survey when individuals rated peers, ICC(1) and ICC(2) were large and excellent, and the model had good internal consistency (total ω = 0.94). For the GLS Survey when referred to the whole crew as the referent, resulting ICCs were large and excellent. McDonald’s omega indicated good internal consistency (ω > 0.70): total ω = 0.91 (see Table 4).

##### Variance decomposition

We conducted a generalizability analysis according to the methods outlined in Cranford et al. (2006) to allow for an overall variance for responses by an individual or crew at a given time to be separated into its components (see Tables 5, 6). Generally, three components other than error accounted for most of the variation: the person, person by day, and person by items on the GLS Survey. The between-person variance indicated that crewmembers have different levels of reported GLS scores across time and survey administrations. The person-by-day indicated that crewmembers had different trajectories of GLS scores over time. The person by GLS score indicated that there were specific effects on the GLS Survey for each crewmember across all days.

**Table 5:** Variance composition of Group Living Skills Survey with individual-level response referents.

	Individual raters’ observations of all crewmembers including self (individual-level ref.)						Individual raters’ observations of peers (individual-level ref.)					
	HERA		SIRIUS-19		SIRIUS-21		HERA		SIRIUS-19	SIRIUS-21		
Source of variance	VC	%	VC	%	VC	%	VC	%	VC	%	VC	%
${\hat{\sigma }}^{2}\mathrm{Day}$	0.00	0.24	0.00	0.00	0.05	2.86	0.00	0.30	0.00	0.00	0.04	2.73
${\hat{\sigma }}^{2}\mathrm{Items}$	0.01	0.48	0.00	0.33	0.24	12.96	0.01	0.77	0.00	0.43	0.06	3.83
${\hat{\sigma }}^{2}\mathrm{Person}$	0.29	25.16	0.29	30.26	0.38	20.20	0.43	30.60	0.32	30.22	0.46	28.25
${\hat{\sigma }}_{\mathrm{Day}*\mathrm{Items}}^{2}$	0.00	0.00	0.00	0.14	0.09	4.64	0.00	0.00	0.00	0.00	0.01	0.67
${\hat{\sigma }}_{\mathrm{Day}*\mathrm{Person}}^{2}$	0.05	4.69	0.17	17.82	0.12	6.24	0.07	5.04	0.22	20.32	0.15	9.53
${\hat{\sigma }}_{\mathrm{Items}*\mathrm{Person}}^{2}$	0.48	41.17	0.25	26.23	0.49	25.94	0.52	36.67	0.27	24.94	0.39	23.80
${\hat{\sigma }}_{\mathrm{Error}}^{2}$	0.33	28.26	0.25	25.23	0.51	27.16	0.38	26.63	0.26	24.10	0.51	31.19
Total	1.16	100	0.97	100	1.89	100	1.41	100	1.07	100	1.62	100

VC, variance component estimate; Ref., referent. *N* = 191 observations in HERA, 62 in SIRIUS-19, and 58 in SIRIUS-21.

**Table 6:** Variance composition of Group Living Skills Survey with crew-level response referents.

Individual raters’ observations of crew (crew-level referent)									Team aggregated ratings (individual- and crew-level referents)			
	HERA C5, C6		SIRIUS-19		SIRIUS-21		ISS		HERA Individual Ref.		HERA Crew Ref.	
Source of variance	VC	%	VC	%	VC	%	VC	%	VC	%	VC	%
${\hat{\sigma }}^{2}\mathrm{Day}$	0.01	1.24	0.02	2.67	0.00	0.00	0.08	7.32	0.00	0.50	0.01	2.59
${\hat{\sigma }}^{2}\mathrm{Items}$	0.01	1.28	0.00	0.26	0.00	0.00	0.06	5.43	0.00	0.13	0.00	1.46
${\hat{\sigma }}^{2}\mathrm{Person}$	0.20	33.99	0.40	46.49	0.24	27.06	0.26	23.56	0.24	46.07	0.15	54.07
${\hat{\sigma }}_{\mathrm{Day}*\mathrm{Items}}^{2}$	0.00	0.00	0.00	0.00	0.00	0.00	0.00	0.00	0.00	0.00	0.00	0.00
${\hat{\sigma }}_{\mathrm{Day}*\mathrm{Person}}^{2}$	0.12	19.77	0.22	25.97	0.14	15.70	0.16	14.45	0.02	4.26	0.03	10.40
${\hat{\sigma }}_{\mathrm{Items}*\mathrm{Person}}^{2}$	0.09	16.13	0.04	4.08	0.10	11.57	0.13	11.73	0.15	29.16	0.05	18.17
${\hat{\sigma }}_{\mathrm{Error}}^{2}$	0.16	27.58	0.18	20.53	0.40	45.66	0.42	37.50	0.10	19.86	0.04	13.31
Total	0.59	100	0.87	100	0.87	100	1.11	100	0.52	100	0.28	100

VC, variance component estimate. *N* = 63 observations in HERA C4, 64 in HERA C5, 64 in HERA C6, 62 in SIRIUS-19, 58 in SIRIUS-21, and 152 on the ISS.

##### Estimation of generalizability coefficients

The variance components were used to generate four reliability coefficients, as shown in Table 7. While GLS seeks reliability regarding change over time given long-duration missions, the other calculations offer further suggestions about overall reliability and insights on administration. Reliability (between persons/team) of measures taken on the same day was very good. Since the *R*_1F_ calculation uses information from all days, it may be interpreted similarly to Cronbach’s α average for GLS (all *R*_1F_ > 0.75, with most >0.92; Cranford et al., 2006). Reliability (between persons/team; *R*_1R_) of measures when persons are measured on different days was moderate to good. Reliability (between persons/team) of an average of measures taken over *K* fixed days was excellent (*R*_KF_ > 0.99). Finally, the reliability of change (within person/team; *R*_C_) was moderate to excellent, indicating that the GLS Survey reliably measured individual or team differences as they changed over time.

**Table 7:** Group living survey generalizability coefficients computed from variance component estimates.

Variance components used	R_1F_ (between)	R_1R_ (between)	R_KF_ (between)	R_C_ (change)
**Individual observations of all individuals including self (individual-level ref.)**				
HERA C4, C5, C6	0.95	0.81	0.99	0.77
SIRIUS-19	0.97	0.63	1.00	0.95
SIRIUS-21	0.96	0.68	1.00	0.87
**Individual observations of peers (individual-level ref.)**				
HERA C4, C5, C6	0.96	0.83	0.99	0.79
SIRIUS-19	0.97	0.60	1.00	0.96
SIRIUS-21	0.97	0.69	1.00	0.90
**Individual observations of crew (crew-level ref.)**				
HERA C5, C6	0.87	0.58	1.03	0.78
SIRIUS-19	0.92	0.59	1.01	0.86
SIRIUS-21	0.76	0.54	1.03	0.63
ISS crew referent	0.78	0.47	1.06	0.66
**Team aggregated ratings**				
HERA C4, C5, C6 (individual-level refs)	0.98	0.89	0.99	0.81
HERA C4, C5, C6 (roommate refs)	0.98	0.90	0.99	0.80
HERA C5, C6 (crew-level refs)	0.96	0.79	1.04	0.80

R_1F_ (between) = Reliability (between persons/team) of measures taken on the same fixed day. R_1R_ (Between) = Reliability (between persons/team) of measures when persons are measured on different days. R_KF_ (Between) = Reliability (between persons/team) of an average of measures taken over K fixed days. R_**C**_ (Change) **=** Reliability of change (within person/team). ISS had several different mission durations across various crews, slight variations in administration intervals between crewmembers, and some crews consisting of only *n* = 1 crewmembers participating in the study (the three *n* = 1 crewmember teams were removed). *N* = 63 observations in HERA C4, 64 in HERA C5, 64 in HERA C6, 62 in SIRIUS-19, 58 in SIRIUS-21, and 152 on the ISS.

#### Factor analysis

Confirmatory factor analysis (CFA) was used to examine the underlying structure of the survey. We ran a two-level CFA to account for the complex and nested nature of the data with individuals nested within teams. Each time point was allowed to be independent, but each observation was clustered/nested within individual and crew. We used the two-level complex maximum-likelihood parameter estimates with standard errors and a chi-square test statistic (when applicable) that are robust to non-normality and non-independence of observations. The MLR standard errors were computed using a sandwich estimator. Mission day was added to the models to account for the temporal nature of the data collection, which occurred multiple times throughout the missions. The results did not meaningfully change; subsequently, mission day was omitted from the final models.

**Figure 1:**
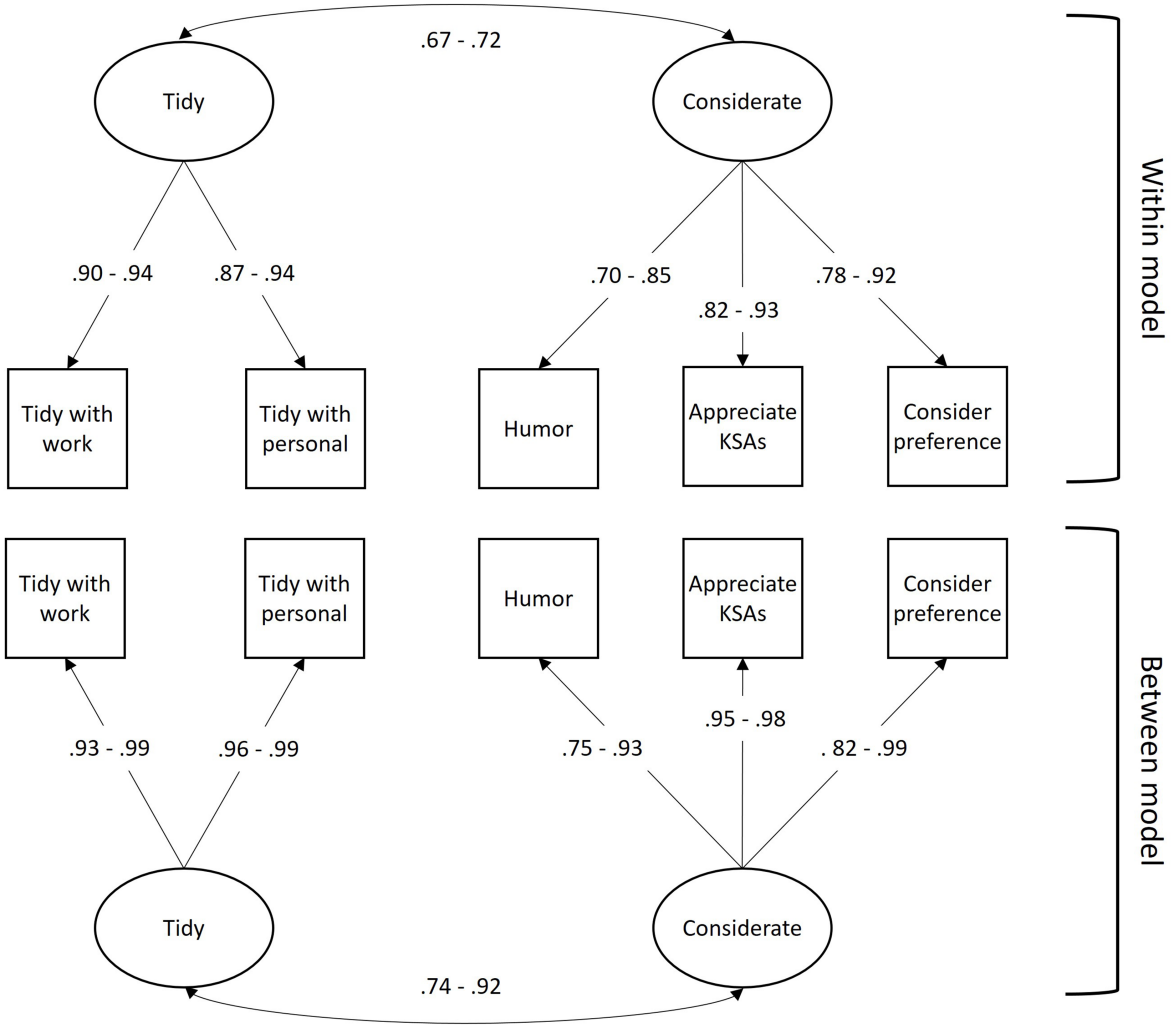
Confirmatory factor analysis results. All parameter estimates are significant (*p* < 0.01). Factor loadings show ranges across all final models with model-specific loadings in Table 8.

We evaluated models according to the model fit guidelines by Mathieu and Taylor (2006). For the model with all individual ratings of other crewmembers and self, the two-factor model (i.e., two factors nested within at the individual level and two factors between at the team level) had excellent comparative fit [*χ*^2^(8) = 20.42, CFI = 0.995, SRMR within = 0.014, SRMR between = 0.014, *p* < 0.01] and good absolute fit (RMSEA = 0.03; Fabrigar et al., 1999). See Figure 1 for the model, Table 8 for specific factor loadings, and Table 9 for model fit indices. For the model of individual ratings of their peers, the model rating the crew, and the model examining individuals’ roommate quality ratings, the same model structure held with excellent fit. We identified the two factors as “Tidy” and “Considerate.”

**Table 8:** Factor loadings for different Group Living Skills Survey models.

	All individual ratings		Peers ratings		Crew referent ratings		Roommate quality ratings	
Factors:	Tidy	Conside-rate	Tidy	Conside-rate	Tidy	Conside-rate	Tidy	Conside-rate
**Within model**								
Clean and tidy with personal items	0.88		0.87		0.94		0.93	
Clean and tidy with work items	0.91		0.91		0.90		0.94	
Uses humor appropriately		0.73		0.76		0.70		0.85
Appreciates others’ knowledge, skills, abilities		0.88		0.90		0.82		0.93
Considerate of others’ preferences		0.90		0.92		0.78		0.92
**Between model**								
Clean and tidy with personal items	0.98		0.98		0.99		0.96	
Clean and tidy with work items	0.95		0.99		0.93		0.99	
Uses humor appropriately		0.85		0.93		0.75		0.92
Appreciates others’ knowledge, skills, abilities		0.96		0.98		0.95		0.98
Considerate of others’ preferences		0.89		0.99		0.82		0.99
**Relationship between estimated latent variables**								
Within model	0.72		0.72		0.70		0.67	
Between model	0.77		0.92		0.74		0.90	
Observations	1809		1,105		398		326	

Table values are standardized factor loadings on intended latent constructs: Tidy and Considerate. All loadings are significant at *p* < 0.01. There were 115 administrations of the Group Living Skills Survey across all crews: four crew administrations for each of the 12 HERA crews, 12 crew administrations in SIRIUS-19, 12 crew administrations in SIRIUS-21, and 43 crew administrations across 10 ISS crews (administrations varied by crew). There were 1,810 observations of all individual and crew referents.

**Table 9:** Confirmatory factor analysis model fit indices.

Models	*df*	χ^2^	CFI	SRMR	RMSEA
All individual ratings	8	20.42	0.995	Within: 0.014 Between: 0.014	0.029
Peers ratings	8	25.68	0.990	Within: 0.019 Between: 0.004	0.045
Crew referent ratings	8	12.55	0.994	Within: 0.015 Between: 0.027	0.038
Roommate quality ratings	8	16.42	0.992	Within: 0.016 Between: 0.005	0.057

df, degrees of freedom; CFI, comparative fit index; SRMR, standardized root-mean-squared residual; RMSEA, root-mean-square error of approximation. All chi-square values (χ^2^) are significant at *p* < 0.01.

##### Test of measurement invariance

We conducted a test of measurement invariance to assess whether the construct, as measured by the GLS Survey, held the same meaning for crewmembers as they completed the survey repeatedly over time. Using all individual ratings and crew referent data from the HERA missions, in which the survey was administered at regular, 10-day intervals four times during the 12 missions, we found support for measurement invariance according to guidelines by Putnick and Bornstein (2016) and Winter and Depaoli (2022). Results suggested metric and scalar invariance of the two-factor model at each time point, matching the model structures illustrated above.

#### Validity analysis

We next examined the relationship between GLS and team cohesion, team performance, and team viability. We conducted generalized mixed models to account for the data collected over time (i.e., mission day) and across campaigns. Our first model included mission day and campaign as predictors of either team cohesion, team viability, or team performance. Our second model added the GLS Survey as a predictor. The predictor utilized was the team-level score of each crewmember’s average rating of the five crew referent items (see Table 1) aggregated by their mission crew (e.g., HERA C4 Mission 1) and the crew mission day, labeled as GLS Crew Score Aggregated in the Tables 10–12. We then looked at the marginal *R*^2^ change between the first and second models to estimate the relationship between GLS and the outcome (e.g., team cohesion, viability, or performance). Results suggested that group living was strongly related to team viability (marginal *R*^2^ change = 0.40), team cohesion (marginal *R*^2^ change = 0.28), social cohesion (marginal *R*^2^ change = 0.25), and moderately related to task cohesion (marginal *R*^2^ change = 0.18). GLS had a small relationship with team performance (marginal *R*^2^ 0.08).

**Table 10:** Model comparisons of Group Living Skills (GLS) Survey scores with team cohesion as the dependent variable.

	Model 1		Model 2	
	GLS crew score aggregated		GLS crew score aggregated	
Predictors	Ests.	CI	Ests.	CI
(Intercept)	6.31***	5.87–6.75	2.91***	2.05–3.77
Mission day	−0.00*	−0.00 to 0.00	−0.00**	−0.00 to −0.00
HERA C5	0.59	−0.06 to 1.24	−0.05	−0.45 to 0.36
HERA C6	−0.05	−0.70 to 0.60	−0.65***	−1.05 to −0.24
ISS	−0.39	−0.94 to 0.16	−0.69***	−1.02 to −0.36
SIRIUS-19	0.26	−0.74 to 1.26	−0.05	−0.60 to 0.51
SIRIUS-21	−0.27	−1.28 to 0.74	−0.75**	−1.32 to −0.17
Team cohesion			0.81***	0.61–1.00
**Random effects**				
σ^2^	0.09		0.06	
τ_00 Crew_	0.17		0.05	
N _Crew_	24		24	
ICC	0.67		0.43	
Observations	115		115	
Marginal *R*^2^/conditional *R*^2^	0.35/0.78		0.64/0.79	

***p < 0.01; **p < 0.05; *p < 0.10.Ests., estimates; CI, confidence intervals. HERA C4 acts as the intercept. Mission day accounts for the longitudinal sampling within each crew.

**Table 11:** Model comparisons of Group Living Skills (GLS) Survey scores with team viability as the dependent variable.

	Model 1		Model 2	
	GLS crew score aggregated		GLS crew score aggregated	
Predictors	Ests.	CI	Ests.	CI
(Intercept)	6.30***	5.88–6.72	3.67***	3.16–4.17
Mission day	−0.00	−0.00 to 0.00	0.00*	−0.00 to 0.00
HERA C5	0.59*	−0.04 to 1.21	0.06	−0.27 to 0.39
HERA C6	−0.05	−0.68 to 0.57	−0.22	−0.53 to 0.09
ISS	−0.36	−0.89 to 0.17	−0.50***	−0.77 to −0.24
SIRIUS-19	0.26	−0.70 to 1.21	0.18	−0.28 to 0.64
SIRIUS-21	−0.29	−1.26 to 0.67	−0.05	−0.52 to 0.42
Team viability			0.47***	0.39–0.55
**Random effects**				
σ^2^	0.09		0.05	
τ_00 Crew_	0.15		0.03	
N _Crew_	24		24	
ICC	0.62		0.38	
Observations	115		115	
Marginal *R*^2^/Conditional *R*^2^	0.342/0.748		0.742/0.841	

***p < 0.01; **p < 0.05; *p < 0.10.Ests., estimates; CI, confidence intervals. HERA C4 acts as the intercept. Mission day accounts for the longitudinal sampling within each crew.

**Table 12:** Model comparisons of Group Living Skills (GLS) Survey scores with team performance as the dependent variable.

	**Model 1**		**Model 2**	
	**GLS crew score aggregated**		**GLS crew score aggregated**	
Predictors	Ests.	CI	Ests.	CI
(Intercept)	6.31***	5.87–6.75	3.46***	2.38–4.54
Mission Day	−0.00*	−0.00 to 0.00	−0.00	−0.00 to 0.00
HERA C5	0.59*	−0.06 to 1.24	0.52*	−0.09 to 1.13
HERA C6	−0.05	−0.70 to 0.60	0.10	−0.52 to 0.71
ISS	−0.39	−0.94 to 0.16	−0.27	−0.78 to 0.25
SIRIUS-19	0.26	−0.74 to 1.26	0.46	−0.49 to 1.41
SIRIUS-21	−0.27	−1.28 to 0.74	−0.14	−1.10 to 0.81
Team performance			0.42***	0.27–0.57
**Random effects**				
σ^2^	0.09		0.06	
τ_00 Crew_	0.17		0.15	
N _Crew_	24		24	
ICC	0.67		0.71	
Observations	115		115	
Marginal *R*^2^/conditional *R*^2^	0.35/0.78		0.43/0.83	

***p < 0.01; **p < 0.05; *p < 0.10.Ests., estimates; CI, confidence intervals. HERA C4 acts as the intercept. Mission day accounts for the longitudinal sampling within each crew.

### Group Living Skills Survey to assess roommate quality

The GLS Survey’s sociometric structure allows for an individual-level assessment of one crewmember’s group living skills by their peers, known as a “Roommate Quality Score.” For these ratings, only HERA and SIRIUS analog crews rated their crewmates at an individual level to allow the calculation of roommate ratings. ISS missions are omitted from the analyses below.

#### Reliability analysis

For the GLS Survey, when each crewmember was rated by their peers on roommate quality, the irrICC package in R was used to calculate Models 1B and 2B (Gwet, 2014) intra-rater reliability coefficients across multiple raters and time points on the target roommate. The resulting ICCs suggest moderate to excellent agreement. McDonald’s omega indicated good internal consistency (ω > 0.70): total ω = 0.96 (see Table 4).

##### Variance decomposition

A generalizability analysis of between-person variance indicated that crewmembers have different levels of reported GLS roommate quality scores across time and survey administrations. The person by day indicated that crewmembers had similar trajectories of GLS roommate quality scores over time. The person by GLS score indicated that there were specific effects on the scores for each crewmember across all days (see Table 13).

**Table 13:** Variance composition of the Group Living Skills Survey of team aggregated ratings with individual roommate referents in the Human Exploration Research Analog (HERA).

Source of variance	VC	%
${\hat{\sigma }}^{2}\mathrm{Day}$	0.00	0.41
${\hat{\sigma }}^{2}\mathrm{Items}$	0.01	1.06
${\hat{\sigma }}^{2}\mathrm{Person}$	0.35	45.07
${\hat{\sigma }}_{\mathrm{Day}*\mathrm{Items}}^{2}$	0.00	0.00
${\hat{\sigma }}_{\mathrm{Day}*\mathrm{Person}}^{2}$	0.03	3.89
${\hat{\sigma }}_{\mathrm{Items}*\mathrm{Person}}^{2}$	0.23	29.54
${\hat{\sigma }}_{\mathrm{Error}}^{2}$	0.16	20.02
Total	0.78	100

VC, variance component estimate. *N* = 63 observations in HERA C4, 64 in HERA C5, and 64 in HERA C6.

##### Estimation of generalizability coefficients

The variance components generated four reliability coefficients listed in Table 7. Results show good to excellent reliability, indicating the GLS Survey reliably measured change over time when crewmembers assessed one particular crewmember.

#### Factor analysis

For the model with all individual ratings of other crewmembers, aggregated to create a roommate quality score for each individual, the two-factor model (i.e., two factors nested within at the individual level and two factors between at the team level) had excellent comparative fit [*χ*^2^(8) = 16.42, CFI = 0.99, SRMR within = 0.016, SRMR between = 0.016 *p* < 0.01] and acceptable absolute fit (RMSEA = 0.057; Fabrigar et al., 1999; Furr, 2017). See Figure 1 for the model, Table 8 for specific factor loadings, and Table 9 for model fit indices.

#### Validity analysis

We examined the relationship between roommate quality and team viability since it was also measured with an individual-level referent and could be calculated as a sociometric measure. We conducted generalized mixed models to account for data being collected over time (i.e., mission day) and across campaigns. The first model included campaign and mission day (time) as a predictor of team viability. Our second model added the GLS Roommate Quality Score. The score was calculated from each crewmember’s average ratings of the five individual referent items (see Table 1) with them as targets (e.g., crewmember-1, -2, -3 scores about crewmember-4). Results indicated a 0.15 marginal *R*^2^ change, suggesting that roommate quality was moderately related to viability (see Table 14).

**Table 14:** Model comparisons of group living skills Group Living Skills (GLS) Survey roommate quality scores with roommate rating of team viability as the dependent variable.

	Individual level			
	Roommate quality score		Roommate quality score	
Predictors	Ests.	CI	Ests.	CI
(Intercept)	6.21***	5.66–6.77	4.45***	4.02–4.88
Mission day	−0.00***	−0.00 to 0.00	−0.00	−0.00 to 0.00
HERA C5	0.72	−0.18 to 1.61	0.37	−0.18 to 0.92
HERA C6	0.05	−0.84 to 0.95	−0.09	−0.64 to 0.46
SIRIUS-19	0.45	−0.94 to 1.85	0.39	−0.46 to 1.24
SIRIUS-21	−0.20	−1.60 to 1.20	−0.12	−0.97 to 0.74
Roommate rating of team viability			0.31***	0.26–0.36
**Random effects**				
σ^2^	0.11		0.09	
τ_00 Person_	0.53		0.32	
τ_00 Crew_	0.33		0.16	
N _Person_	60		60	
N _Crew_	14		14	
Observations	326		326	
Marginal *R*^2^/conditional *R*^2^	0.599/NA		0.750/NA	

***p < 0.01; **p < 0.05; *p < 0.10.Ests., estimates; CI, confidence intervals. HERA C4 acts as the intercept. Mission day accounts for the longitudinal sampling within each crew.

## Discussion and future directions

The purpose of our research was to develop and provide initial reliability and validity evidence of a new GLS measure. Our results suggest the five-item measure (Table 1) is reliable, valid, and operationally feasible, filling a gap in the literature to assess this unique team-oriented skill area. Furthermore, our results indicate this measure can provide a team-level assessment of group living skills or a peer rating of roommate quality. Few work environments couple living and working together with colleagues for a series of weeks, let alone months or years. In such extreme work environments, the line between personal and work life is blurred (Landon and Paoletti, 2021). We define group living skills as the capability to consider and accommodate others’ needs and personal preferences to maintain cohesion when living together. When placed in the context of work, this consideration and accommodation of others must be for both interpersonal- and work-related issues.

The analyses employed for this measure development accounted for a complex data collection protocol. Individual crewmembers were nested within crews. There were three habitats (i.e., HERA, SIRIUS, ISS) encompassing multiple unique mission conditions such that there were six distinct extreme environments (i.e., HERA campaigns 4, 5, 6; SIRIUS-19, SIRIUS-21, ISS). Data were collected over time (i.e., 45- to 240-day missions) with a repeated measures design. The survey was examined as a team measure and as a sociometric measure. Across these complexities, our analyses demonstrated that the GLS Survey is a valid measure to assess group living skills for a team or any sort of roommate. We also demonstrated that the GLS Survey is reliable over time; it detects differences between individuals and crews and enables the monitoring of a team’s group living skills over time. Reliability analyses showed differences between various referents and environments; for example, the error components in the variance decomposition of the crew referent scale resulted in lower *R*_1R_ coefficients. We statistically accounted for and tested for compatibility with the overt scale refinements across the years of data collection, but there may have been subtle effects leading to higher noise. The cause of the residual errors is somewhat unclear, and more data are needed. For factor analysis, the GLS Survey demonstrated consistent factor structure at different levels and for different types of administrations, including for the roommate quality sociometric approach. The factor analysis suggests the following two factors: tidy and considerate. Future research could collect data in more controlled environments when possible, reducing noise from some aspects of our complex data collection environments and inserting more measures to investigate divergent validity. In general, more data collection is needed to fully understand this construct and the effects of group living skills on individuals and teams in various environments and configurations (e.g., larger teams, less isolated teams, rotating teams, and teams with differing compositions of roles and individual characteristics).

Researchers can employ the GLS Survey to capture an otherwise neglected aspect of teams working and living in isolated and extreme environments. Application in other extreme environments such as military bases and forward operating bases, Antarctic stations, oil rigs, remote search-and-rescue, firefighters, and transoceanic shipping may enable further understanding of how group living skills influence team cohesion and performance. Other less extreme environments may also benefit from research with this measure if they also have teams living and working together for some length of time. These may include camp counselors, cruise ship employees, traveling entertainment troupes, or even typical roommate situations at college or otherwise. The items were written to be applicable to any type of environment in which people live with those with whom they work, and more research in a variety of applicable environments is needed. Our high-performing samples (i.e., astronauts or astronaut-like individuals) tend to endorse higher values on team dynamics measures, often because they are selected and trained to have a high degree of team orientation and team skills. This introduces range restriction, but the GLS item endorsements did capture some datapoints at the lower to middle end of the scales, which varied between crews and individuals. In other words, the scale was sensitive even in this extremely high-performing population. Future research with other samples is warranted. Similarly, it may be of interest for researchers to use the sociometric roommate quality approach to the GLS Survey to understand the relationships between how a person’s group living skills can affect team dynamics, behavioral health, and well-being. We also collected open-ended responses on the GLS Survey, asking crewmembers to elaborate as desired on any of the ratings they provided on the five-item survey. The analysis of these comments is beyond the scope of this article, but preliminary review suggests there is likely another benefit of assessing team dynamics with the GLS Survey in that it detects smaller frictions or tensions within a group that were not captured on a conflict scale. For example, one of the crews in this study reported no conflicts, but their GLS Survey and the GLS comments suggest friction among crewmembers, which can also disrupt cohesion and performance (Marcinkowski et al., 2021).

Practitioners supporting groups and teams who live and work together may benefit from using the measure for selection, team composition, training, and developmental feedback by operational psychologists and team leaders. During selection and training, the GLS Survey can be used to identify individuals particularly well-suited for such extreme living and working situations by considering self-reports, ratings of others in a selection or training simulation (i.e., other applicants or trainees or expert observers), or ratings from others that have prior experience living with the referent. Situational judgment tests are set in a workplace living situation, and the GLS Survey is utilized as a basis for rating responses to test items. This may be used to assess group living skills when high-fidelity simulations are not feasible. Trainers at other organizations may use the behavioral markers to customize group living skills training for their workforce, using the information in this work as guidance for specific behaviors on which to train, rate, and provide feedback. Astronauts develop and practice these skills during various “field trips” to train for geology training excursions, mission simulations, and wilderness backpacking trips that may last several days. If possible, it is recommended that training should occur in a context similar to the mission to elicit relevant behaviors. This is a unique skillset, especially compared to more typical work situations, so context is important. During a mission, the use of the individual-level referent approach allows for a fine-grained understanding of the interpersonal dynamics of a crew, which can inform targeted training and feedback to individuals or the team along with the behaviors in the items. Individual-level ratings can be aggregated to the team level to achieve a team-level look at GLS scores, with and without the influence of self-ratings. Our TOST analysis found the aggregation of all individual ratings, including self-ratings using individual-level referents, was not significantly different than directly assessing crew with the crew-level referent. Future research should examine the extent to which self-ratings align with an individual’s ratings of their peers (which may indicate a shared mental model) and with their peers’ roommate quality score for that individual (which may indicate self-awareness). In time-constrained situations such as on the ISS, the survey may be deployed only with the crew-level referent for a quick understanding of the crew’s current level of group living skill execution. Again, this enables the strategic implementation of crew countermeasures to support crews as their team dynamics change over time.

Finally, we recommend a regular, repeated data collection protocol for the GLS Survey in both research and applied settings. The frequency of these administrations ranged from every 10 days in a 45-day analog mission (i.e., HERA) to every 20 days for multi-month analog missions of 120–240 days (i.e., SIRIUS) to monthly for an applied operational environment (i.e., the ISS). We recommend at least monthly administrations at a minimum for a long-duration mission. This balances a need for monitoring the team over time and avoiding survey fatigue or burden in an operational environment; however, we do encourage a greater frequency if possible. We also recommend that all crewmembers complete the GLS Survey on the same day at the same time to capture the current team-level dynamics. Additional sampling around a significant change in living situations or events may warrant extra administrations (e.g., a crew rotation on the ISS may necessitate additional sampling 2 weeks before and after the change). We hope that researchers and practitioners alike find value in using the GLS Survey to measure this unique construct of teams living in extreme work environments.

## Data availability statement

The datasets presented in this article are not readily available because data collected as part of NASA-funded research is archived in the NASA Life Sciences Data Archive. Requests to access the datasets should be directed to https://nlsp.nasa.gov/explore/lsdahome.

## Ethics statement

The studies involving humans were approved by NASA Institutional Review Board. The studies were conducted in accordance with the local legislation and institutional requirements. The participants provided their written informed consent to participate in this study.

## Author contributions

LBL: Conceptualization, Data curation, Formal analysis, Investigation, Methodology, Project administration, Supervision, Validation, Visualization, Writing – original draft. JCWM: Data curation, Formal analysis, Investigation, Methodology, Validation, Writing – original draft. STB: Formal analysis, Investigation, Methodology, Project administration, Supervision, Validation, Writing – original draft. PGR: Conceptualization, Data curation, Funding acquisition, Investigation, Methodology, Project administration, Supervision, Writing – review & editing.

## Appendix A. All other measures

**Team Cohesion** (Kozlowski, 2015).

*Instructions*: Answer the questions below with regard to what happened today. (*anchors*: 1 = Strongly Disagree, 4 = Neutral, 7 = Strongly Agree).

**Social Cohesion**

1. Our team members cared about each other.**
2. Our team members had good relationships with each other.
3. Our team members enjoyed each other’s company.

**Task Cohesion**

1. Our team was unified in its task focus.**
2. Our team had a shared sense of task importance.
3. Our team was committed to our team’s task(s).

**Team Performance** (Tannenbaum and Mathieu, 2015).

*Instructions*: Answer the questions below with regard to what happened today. (*anchors*: 1 = Not at All, 4 = To a Moderate Extent, 7 = To a Very Great Extent).

1. To what extent did the crew accomplish its primary goals today?
2. To what extent were the important tasks for today done in a high-quality fashion?
3. Taking everything into consideration, to what extent did the crew perform well today?**
4. To what extent were the important tasks for today done in a timely fashion?

**Team Viability** (adapted from Vinokhodova et al., 2012).

**Individual referent.**

*Instructions*: Please rate your level of agreement with the following statements about [*selected teammate*]: (*anchors*: 1 = Strongly Disagree, 4 = Neutral, 7 = Strongly Agree).

1. If I were doing another mission, I would want [selected teammate] as a crewmate.

**Crew referent.**

*Instructions*: In your experience, please rate your level of agreement with the following statements for your entire crew: (*anchors*: 1 = Strongly Disagree, 4 = Neutral, 7 = Strongly Agree).

1. If I were doing another mission, I would want this exact same crew.**

**Indicates items used on the ISS short-form surveys.
